# Cathodoluminescence of green fluorescent protein exhibits the redshifted spectrum and the robustness

**DOI:** 10.1038/s41598-020-74367-4

**Published:** 2020-10-15

**Authors:** Keiichirou Akiba, Katsuyuki Tamehiro, Koki Matsui, Hayata Ikegami, Hiroki Minoda

**Affiliations:** Department of Applied Physics, Tokyo University of Agriculture and Technology, 2-24-16, Nakacho, Koganei, Tokyo 184-8588 Japan

**Keywords:** Biological physics, Biological fluorescence, Electron microscopy

## Abstract

Green fluorescent protein (GFP) and its variants are an essential tool for visualizing functional units in biomaterials. This is achieved by the fascinating optical properties of them. Here, we report novel optical properties of enhanced GFP (EGFP), which is one of widely used engineered variants of the wild-type GFP. We study the electron-beam-induced luminescence, which is known as cathodoluminescence (CL), using the hybrid light and transmission electron microscope. Surprisingly, even from the same specimen, we observe a completely different dependences of the fluorescence and CL on the electron beam irradiation. Since light emission is normally independent of whether an electron is excited to the upper level by light or by electron beam, this difference is quite peculiar. We conclude that the electron beam irradiation causes the local generation of a new redshifted form of EGFP and CL is preferentially emitted from it. In addition, we also find that the redshifted form is rather robust to electron bombardment. These remarkable properties can be utilized for three-dimensional reconstruction without electron staining in focused ion beam/scanning electron microscopy technology and provide significant potential for simultaneously observing the functional information specified by super-resolution CL imaging and the structural information at the molecular level obtained by electron microscope.

## Introduction

Green fluorescent protein (GFP) exhibits bright green fluorescence at room temperature without exogenous cofactor^1^ and the use of it and its variants as a tool for microscopy has revolutionized in the live cell imaging^2^. Moreover, they have brought us the intriguing photophysical and/or photochemical phenomena^3^ such as photoswitching, photoactivation, and photoconversion. These also play a prominent role in visualizing the biological events. These kinds of fascinating and applicable optical properties of GFP are provided by the light/laser illumination. In the context of luminescence, it is noted that electron beam irradiation on a luminescent material causes the emission of photons i.e., cathodoluminescence (CL). Even though biological materials usually suffer the considerable damage from electron bombardment^4^, CL of GFP has been clearly observed so far^5,6^. This opens up the next phase in bioimaging using GFP, since a convergent electron beam can readily induce the CL in tiny region, which is small enough to overcome the diffraction limit of light due to the wave nature of electron. However, the effect of electron irradiation and the CL property of GFP have not been fully investigated. Therefore, it is important to clarify them from both fundamental and applicative aspects.

In this paper, we perform a spectroscopic study on the CL of enhanced GFP (EGFP), which is one of widely used engineered variants of the wild-type GFP, together with its fluorescence, i.e., photoluminescence (PL) before/after electron irradiation. We used fluorescence-electron hybrid microscope, which is a transmission electron microscope (TEM) integrated with a home-built fluorescence light microscope. We, here, observe the two surprising features. The spectra of CL and PL are clearly different even when the emitted light is collected from the same ensemble of EGFP. Note that once an electron is excited to an upper level of the excited state, it relaxes to the lowest level of the excited state and then emits a photon, which property is independent of how to excite the electron to the upper level. Furthermore, the intensity of CL is almost unchanged. This means that the CL of EGFP is tolerant of the electron bombardment.

These striking features provide not only CL microscopy of biomaterials but also significant potential that unveil the relationship between the structure and function of biological specimen. As is known in the success of cryo-electron microscopy, the electron microscope visualizes the structure at the molecular level^7^. In contrast, the GFP labels functional units in cells or tissues with the gene expression technology and thus the tolerant CL can achieve the light imaging of functional units over diffraction limit. Especially in the beneficial method of the correlative light and electron microscopy (CLEM)^8^, the CL availability eliminates the viewing field gap between light and electron image and then constructs the straightforward combination between them. This leads to next phase of the high resolution bioimaging that the function of biological specimens with sufficient resolution is obtained by CL imaging while the corresponding molecular structure is simultaneously observed with an electron microscope.

## Results and Discussion

**Figure 1:**
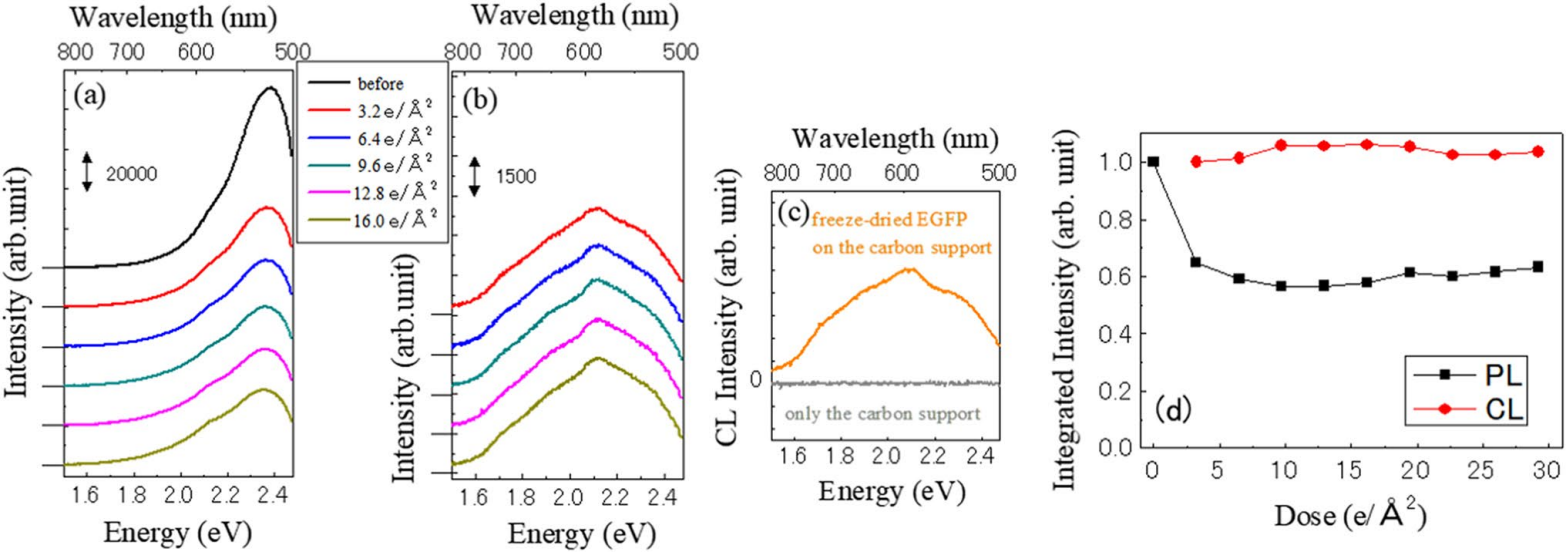
Typical spectra of (**a**) PL before/after electron beam irradiation and (**b**) CL of EGFP. The curves are offset for clarity. The upper horizontal axis indicates the light wavelength corresponding to the photon energy. The graph legends indicate the amount of the electron dose. The electron dose values for the CL spectra include the electron injection to collect emission photons (3.2 e/Å^2^). The PL and CL spectra were obtained alternately. (**c**) Typical CL spectra of only freeze-dried EGFP (purity ≥ 97%) on the carbon support and only the carbon support. This strongly supports that the luminescence comes from EGFP. (**d**) Electron dose dependency of the integrated PL and CL intensity. All data were normalized so that the initial values of the integrated PL and CL intensity were equal to unity. The data series to make (**d**) are the same as (**a**).

Figure 1 shows spectra of (a) PL (fluorescence) before/after electron beam irradiation and (b) CL of an EGFP sample. In this experiment, the efficiency that the electron transmitted through sample is 6% and the dose rate is 0.64 e/Å^2^/s. With the increasing electron dose in (a), the PL intensity decreases and the lower energy shoulder (~ 2.1 eV or 590 nm) relatively grows (a peak appears). Subsequently, there is little change in the PL spectra. In contrast to the PL, both the intensity and shape of the CL spectra (shown in Fig. 1b) are almost the same at different levels of electron irradiation. While the PL spectrum before electron irradiation attains a normal EGFP spectrum, the CL spectra are clearly different from those of PL and the peak appears at 2.1 eV (Fig. 1b). Although the electron irradiation seems to destroy some components of EGFP and change the spectra, the PL spectra after electron beam exposure also differs from the CL spectra, where we acquired the PL and CL spectra alternately.

We could consider this behavior as if there were two different samples. However, both sets of PL and CL results are collected from the same ensemble of EGFP. It should be noted that CL spectroscopy is well-established technique to probe various kinds of materials. Except for CL, surface plasmon radiation and transition radiation are known to be light generation induced by electron beam irradiation. These phenomena should not be pronounced in our experiment. Indeed, when electron beam hit Cu grid of TEM, any spectrum was not observed under our experimental conditions.

We carefully checked whether the substances around EGFP emit concerned photons due to the electron irradiation or not. Figure 1c shows CL spectra of freeze-dried EGFP (purity ≥ 97%) without any buffer solution on the carbon support and only the carbon support. The carbon support is found to be non-luminescent. This means that the observed CL in (c) just comes from EGFP. The CL spectral shape in (b) is quite similar to that in (c), indicating the observed CL in (b) also comes from EGFP. Although the excitation process of photon emission is different between PL and CL, the photon emission process is generally independent of how the electrons are excited to the upper level; an excited electron relaxes to the lowest energy level of the excited state and emits a photon. The different observation between PL and CL has been known in the research on a diamond nitrogen-vacancy (NV) centre. While PL shows the emission from both negatively-charged and neutral states, CL exhibits the emission only from neutral state. This fact is also surprising and its mechanism was recently studied^9^. Here, we emphasize the difference between diamond NV centre and GFP. The initial PL spectrum of GFP does not include the CL spectrum, while the PL spectrum of the NV centre always includes the CL spectrum.

As described above, the observed behavior that was completely different between PL and CL is quite peculiar.

First, we consider what is meant by the CL spectrum. It is known for GFP that three forms of the chromophore, i.e., the neutral A-form, anionic B-form, and intermediated I-form, are involved in green fluorescence^10–12^, and a recent investigation indicates that these forms also exist in EGFP^13^. However, the energy of the observed CL peak is lower than the peaks of all of these forms. The single-molecule spectroscopy study of one of GFP variants shows that a small number of redshifted forms exist in the ensemble^14^. If the electron beam preferentially excites one of such forms in our prepared sample, we could understand the CL spectrum. However, the luminescent excitation in CL covers all of the optical radiation energy range because the initial energy of the injected electron is fairly huge. In this case, the emission observed in PL must appear in CL. Since we cannot excite only the redshifted forms by electron irradiation, we should observe both normal fluorescence and redshifted peaks. We are unlikely to observe only a redshifted peak in CL. Here, we suggest that a novel form of EGFP, which emits a redshifted fluorescence, is generated by the electron beam irradiation. When an electron accelerated with high voltage is injected to the material, secondary electrons escape from the latter. Since the material loses electrons, this can be considered as an oxidation process. In previous investigations^13,15,16^, the excited state of EGFP in the oxidant environment induces the generation of the redshifted form of the chromophore, and we believe that a kind of similar scenario occurs due to the electron beam irradiation. The relatively broadened CL spectrum could be understood by the effect of various electron beam damages around the chromophore.

**Figure 2:**
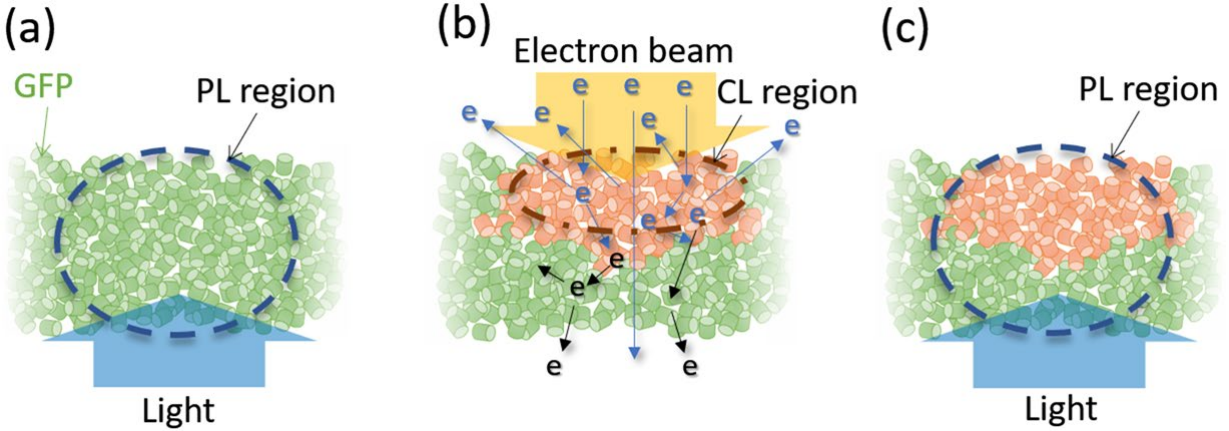
Schematics of the physical picture (**a**) before, (**b**) during, and (**c**) after electron beam irradiation. A small circular cylinder represents a single GFP. The violet-blue light illuminating from the bottom side excites the PL and the electron beam irradiation from the upper side excites the CL. The PL (CL) region corresponds to the excitation of the PL (CL) emission region. The PL and CL are collected from the region where the electrons pass through.

On the basis of our consideration that the redshifted form of EGFP is generated by electron beam irradiation, we assume the following physical picture in order to explain the observed peculiar behavior. A schematic is shown in Fig. 2. In the actual situation, the EGFP ensemble should be partly aggregated and the sample should be inhomogeneous with its density and thickness. Although this inhomogeneity can introduce somewhat moderate change, the use of a homogeneous sample in Fig. 2 simplifies the situation to help understand the essential point without difficulty. We consider the region through which the electrons pass as the light collecting area. Before the electron irradiation, the general fluorescence of EGFP is of course obtained, where the PL emission region corresponds to the light illuminating area and excitation light is injected from the downside (Fig. 2a). When the electron beam is irradiated from the upside, the majority of the EGFP in the upper region transforms to the redshifted form (Fig. 2b), as previously described. When the injected electrons penetrate the EGFP ensemble, they generate a lot of secondary electrons and lose their energy due to the electron scattering process. The secondary electrons generated in the specimen cannot run a long distance due to their small energy, resulting in their escape from the upper region only. According to our assumption concerning the oxidative generation of the redshifted form, this is the reason why only the EGFP in the upper region is transformed. Since the secondary electrons mainly cause CL, the spatial distribution of them affects the CL emission region. This accounts for the fact that only CL exhibits the spectrum of the redshifted form. Assuming the homogeneous electron transmission of 6% through the sample, a half of the primary electrons is lost in one-quarter of thickness from the upside. The relatively large numbers of the secondary electrons are considered to exist in the upper region and thus predominantly excite the redshifted EGFP (Fig. 2b). The actual inhomogeneity of the EGFP ensemble can also contribute the enhancement of the local emission of CL because there is a possibility that electrons cannot be transmitted in a certain region. The limited emission region can also explain why the CL presents much lower intensity than the PL in Fig. 1. After the electron irradiation, the PL emission region includes the original and redshifted EGFP (Fig. 2c, in which the PL consists of these spectra), thus the shape of the PL spectrum is changed. In the next electron beam irradiation, the electron scattering process is almost the same as Fig. 2b. The redshifted form can be gradually generated (the region of the redshifted EGFP slightly grows) and the original EGFP is degraded while the redshifted EGFP is not so influenced. Indeed, this causes the peak of the redshifted form (2.1 eV) to become more pronounced in Fig. 1a.

**Figure 3:**
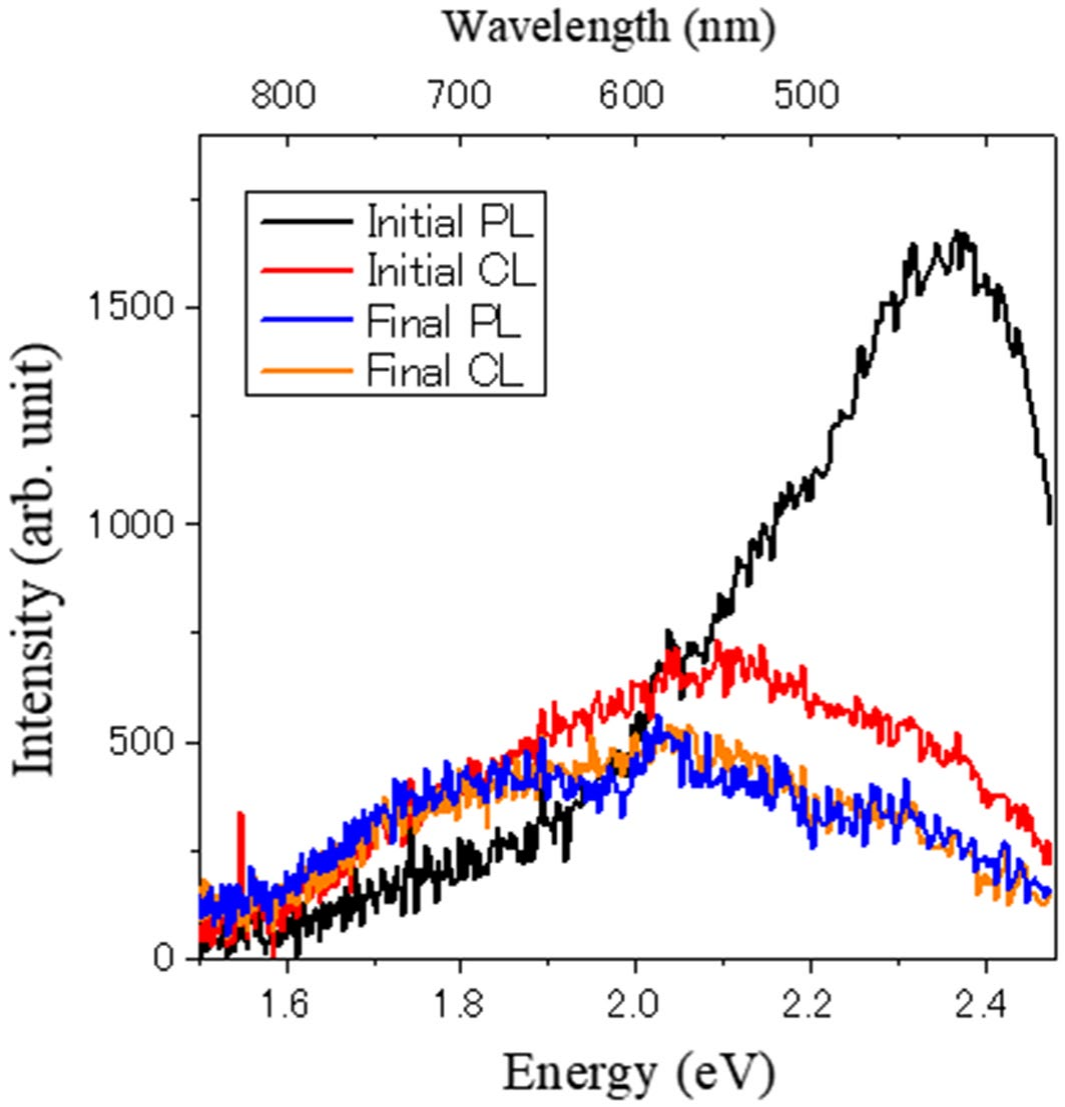
PL and CL Spectra for the thinner sample. The electron transmission was around 70%. The upper horizontal axis indicates the light wavelength corresponding to the photon energy.

To confirm our assumption, we measure the PL and CL using a thinner sample (Fig. 3). The overall electron transmission of the irradiated area is around 70%. This results in less than one eighth of the thickness compared with the sample in Fig. 1 by assuming the density of sample as homogeneous. Here, it is difficult to estimate the density of EGFP sample and we cannot estimate the values of thickness. Since the thinner sample corresponds to the only upper region of the schematic in Fig. 2, the PL is expected to be identical to the CL after sufficient electron beam irradiation. The initial PL spectrum (before electron irradiation) is qualitatively the same as Fig. 1a. The slight difference of the lower energy intensity can be explained by the inhomogeneity of a small ensemble. The initial CL is also the same as Fig. 1b. After an electron dose of 700 e/Å^2^, the PL spectrum coincides with the CL spectrum (the final PL and CL spectra in Fig. 3). This fact demonstrates that our assumption is correct. The slight difference between the initial and final CL is understood by the electron beam damage of the redshifted EGFP due to the huge electron dose. It is noted that CL intensity is almost the same as PL intensity in Fig. 3. This means that the CL emission is not weak compared to the PL and is available for super resolution bioimaging. The large intensity difference between CL and PL in Fig. 1 is due to the difference of the emission volume as we discussed above.

Next, we discuss the electron irradiation damage to EGFP. The typical example of the electron dose dependences of the integrated PL and CL intensity are shown in Fig. 1d. Integrated PL intensity decreases just after initial electron beam irradiation, while the integrated CL intensity exhibits almost constant in this electron dose range. Here, even when the amount of the initial dose was decreased, we observed the initial decrease in integrated PL intensity.

The CL spectra exhibit almost the same shape while the PL spectra changes their shape by the electron beam irradiation (see Fig. 1a,b). As we already discussed, the change in the spectral shape is due to the generation of the redshifted form. Thus, the integrated PL intensity cannot trace the amount of one form of EGFP. The relatively large change in the integrated PL intensity by first electron dose means that once small amount of electron dose is applied to GFP, lots of the redshifted form are generated. The number of a secondary electron to observe CL could be enough to transform from the original EGFP to the redshifted one. This could be one reason for the quick appearance of the redshifted form.

Since the shape of the CL spectra hardly change in the experiment, the integrated CL intensity directly reflects the surviving amount of the redshifted EGFP. We, here, performed lots of series of experiments to extract quantitative values for electron beam damage. However, we cannot clearly obtain single characteristic value for the simple exponential decay function under the fixed experimental condition. This means that an experimental parameter that we cannot precisely control influences estimation on characteristic values. Therefore, the discussion on the electron irradiation damage to EGFP should remain relatively qualitative here. We can discuss the electron beam damage to the redshifted form from the change in the integrated CL intensity.

Since most amino-acid molecules are destroyed at several e/Å^2^^17^, we can say that the redshifted EGFP is stronger from Fig. 1d. In addition, the CL decay in coronene has been reported to be a factor of 20 lower than for the destruction of crystallinity and a factor of 200 lower than for the destruction of the molecular structure, which means that CL possesses the high sensitivity for the electron beam damage; the reported value of characteristic dose of the CL intensity for coronene was 2.7 e/Å^2^ at 100 keV^18^. While the CL intensity of coronene can be expected to be 16%, 2%, and 0.1% of the initial value at 5, 10, and 20 e/Å^2^, respectively, the CL intensity of the redshifted EGFP remains almost unchanged even at 30 e/Å^2^ in Fig. 1d. Therefore, we figure out that the emergent redshifted EGFP is rather robust to the electron beam irradiation, even though we cannot estimate the damage quantitatively here and a rough estimation of electron damage shows fluctuation of an order of magnitude. Furthermore, if we only consider GFP usage of luminescent label for CL microscopy and the change in spectral shape does not matter, we have already obtained good value, which is more than 70% surviving after huge electron dose of 700 e/Å^2^ in Fig. 3. This would be due to that a certain component of the redshifted EGFP is much stronger against the electron irradiation and survives after the deterioration of the relatively weak component.

The integrated PL intensity shows the initial large decrease and subsequently almost constant. Since the initial decreases is associated with the change in the spectral shape, the latter constant could reflect the amount of the redshifted and the resultant original EGFP. This can suggest the original EGFP is also robust.

As describe above, we strongly believe that our results at least show the robustness of the redshifted EGFP. Although we cannot deduce the reason of its robustness scientifically, one possible consideration may be that β-barrel structure enclosing the chromophores can protect them.

In conclusion, we studied the CL of EGFP by using the hybrid fluorescence and electron microscope. We found that the behaviors of CL and PL observed from the same ensemble of EGFP were not identical. The CL spectrum was redshifted from the original EGFP spectrum, which was explained by the generation of a novel form of EGFP due to the electron beam irradiation. The surprising difference between CL and PL was explained as the consequence of the local generation of the novel form and preferential local emission of CL. We also found that the novel redshifted EGFP was rather robust to the electron bombardment. In particular, we observed more than 70% survival of CL intensity at huge electron dose of 700 e/Å^2^. This property is what requires in the challenging CL imaging of the organic materials. Even though a further investigation is required on the detailed mechanism of the generation of the novel EGFP form and its robustness, our findings enables super-resolution CL imaging of the function of biological specimens. Since the CL of EGFP emits from the quite shallow region, we suggest a novel application for focused ion beam/scanning electron microscopy (FIB/SEM) technology^19^. The relatively low acceleration voltage of SEM compared with TEM limits the thickness of CL emission (secondary electron generation) region to less than several tens of nanometers. Therefore, a careful choice of acceleration voltage and treatment of the specimen can visualize a GFP-labeled functional unit with less than 10 nm thickness by using CL. This achieves super-resolution three-dimensional reconstruction without electron staining in FIB/SEM technology. Moreover, combining super-resolution CL imaging with recently developed phase contrast scanning transmission electron imaging using an environmental cell^20,21^ opens up the possibility of simultaneous observation of light and electron images with extreme spatial resolution without chemical staining.

## Methods

### Sample preparation

The EGFP samples we used were EGFP (EGFP plasmid purchased from Addgene) in 10 mM Tris–EDTA buffer solution, STA-201 purchased from Cell Biolabs (buffer solution: 1 × PBS), and freeze dried EGFP purchased from BioVision. These three types of EGFP sample basically exhibited the same optical properties in our experiments. The EGFP solution was added to the TEM grid made of copper with a 10 nm carbon film support and was dried in ambient air. The dried GFP should be ununiform and the density of it cannot be obtained. A specimen for TEM is small: the grid diameter is 2 mm and the diameter of the observation windows in it is 100 μm. This gave rise to the difficulty to determine the sample details. We cannot identify the electron-irradiated region in the sample after measurement.

### Fluorescence-electron hybrid microscopy and spectroscopic measurement

**Figure 4:**
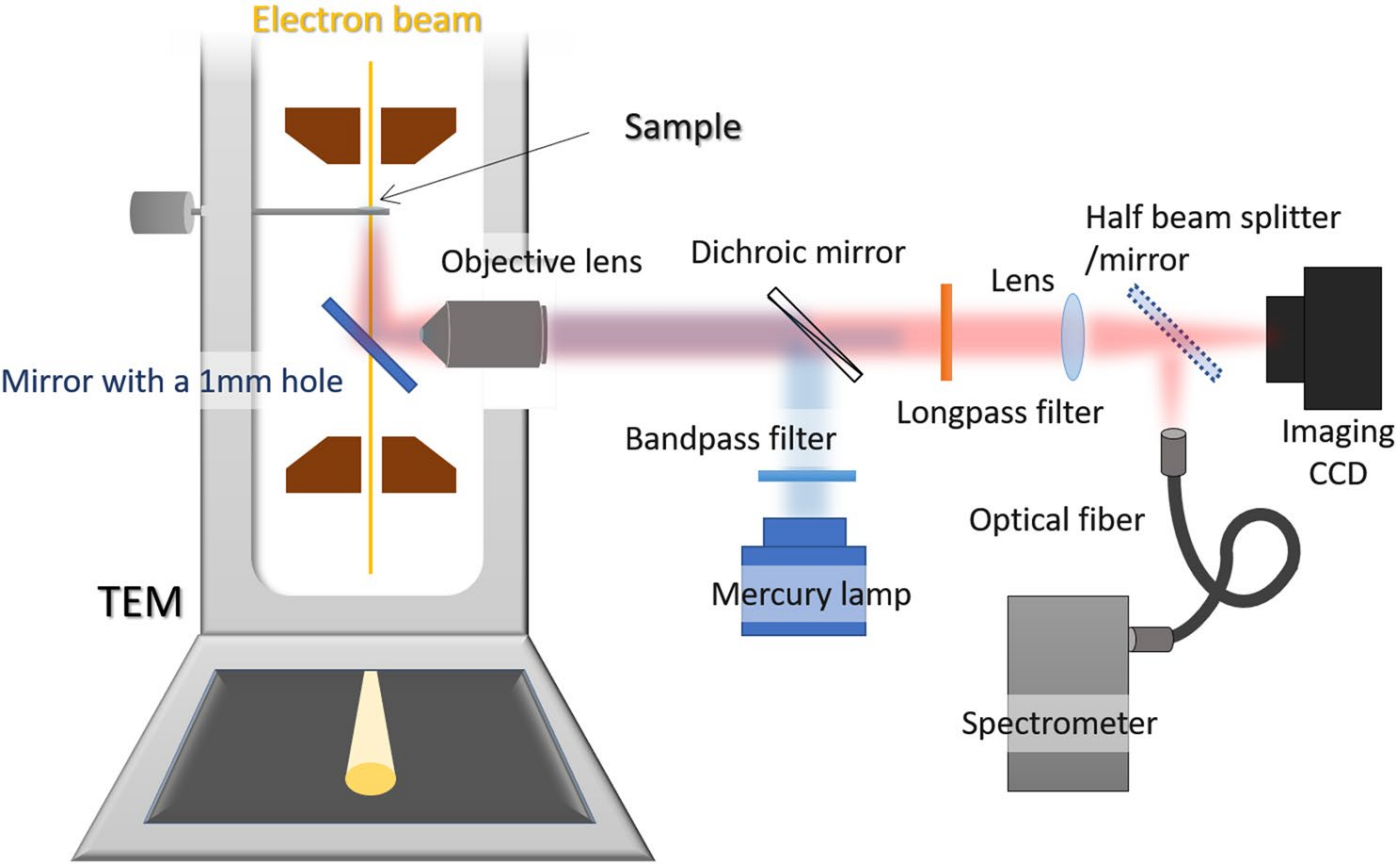
Schematics of the experimental setup. The details of the hybrid light and electron microscope we used in the experiments.

The hybrid microscope is a commercial TEM (JEM-2010, JEOL) into which is inserted a home-built fluorescence light microscope. The details have been reported in our previous work^6,22^. The schematic of the experimental setup is shown in Fig. 4. TEM was operated at 200 kV. Optical excitation was performed via a mercury lamp with an excitation filter of 400–480 nm (SHPF-25c-492, SIGMAKOKI) through a carbon film support. The emitted light from the EGFP samples was collected through a carbon film support and an emission long pass filter (FELH500, Thorlabs). This imposes the limitation on the observed spectral range. Especially, the emission peak of A-form (~ 450 nm) cannot be measured. However, this fluorescence is quite week and one-order smaller than that of B-form^23^, which means that the emission of A-form is negligible.

The reason why we set this spectral range is as follows. To compare PL with CL, excitation energy range for PL should be as close as that for CL, which covers all visible energy. The excitation and collection were divided by a dichroic beam splitter. The electron irradiation area was 2.2 × 10^2^ μm^2^. The light collection area for the optical spectroscopy was within the electron irradiation area, where the light microscope images enabled us to check these areas.

## Data Availability

The data related to this study are available from the corresponding author upon reasonable request.

## References

[1] Chalfie M, Tu Y, Euskirchen G, Ward WW, Prasher DC (1994). Green fluorescent protein as a marker for gene expression. Science.

[2] Tsien RY (1998). The green fluorescence protein. Annu. Rev. Biochem..

[3] Jung G (2013). Fluorescent Protein I: From Understanding to Design.

[4] Egerton RF, Li P, Malac M (2004). Radiation damage in the TEM and SEM. Micron.

[5] Fisher PJ, Wessels WS, Dietz AB, Prendergast FG (2008). Enhanced biological cathodoluminescence. Opt. Commun..

[6] Nagayama K, Onuma T, Ueno R, Tamehiro K, Minoda H (2016). Cathodoluminescence and electron-induced fluorescence enhancement of enhanced green fluorescent protein. J. Phys. Chem. B.

[7] Callaway E (2015). The revolution will not be crystallized: a new method sweeps through structural biology. Nature.

[8] de Boer P, Hoogenboom JP, Giepmans BNG (2015). Correlated light and electron microscopy: ultrastructure lights up!. Nat. Methods.

[9] Solà-Garcia M, Meuretm S, Coenen T, Polman A (2020). Electron-induced state conversion in diamond NV centers measured with pump-probe cathodoluminescence spectroscopy. ACS Photonics.

[10] Creemers TMH, Lock AJ, Subramanian V, Jovin TM, Völker S (1999). Three photoconvertible forms of green fluorescent protein identified by spectral hole-burning. Nat. Struct. Biol..

[11] Creemers TMH, Lock AJ, Subramanian V, Jovin TM, Völker S (2000). Photophysics and optical switching in green fluorescent protein mutants. Proc. Natl. Acad. Sci. U.S.A..

[12] Chattoraj M, King BA, Bublitz GU, Boxer SG (1996). Ultra-fast excited state dynamics in green fluorescent protein: multiple states and proton transfer. Proc. Natl. Acad. Sci. U.S.A..

[13] Saha R (2013). Light driven ultrafast electron transfer in oxidative redding of green fluorescent proteins. Sci. Rep..

[14] Blum C, Meixner AJ, Subramaniam V (2004). Room temperature spectrally resolved single-molecule spectroscopy reveals new spectral forms and photophysical versatility of *Aequorea* green fluorescent protein variants. Biophys. J..

[15] Elowitz MB, Surette MG, Wolf PE, Stock J, Leibler S (1997). Photoactivation turns green florescent protein red. Curr. Biol..

[16] Bogdanov A, Mishin A, Yampolsky I (2009). Green fluorescent proteins are light-induced electron donors. Nat. Chem. Biol..

[17] Reimer L, Kohl H (2008). Transmission Electron Microscopy.

[18] Li P, Egerton RF (2004). Radiation damage in coronene, rubrene and p-terphenyl, measured for incident electrons of kinetic energy between 100 and 200 keV. Ultramicroscopy.

[19] Knott G, Marchman H, Wall D, Lich B (2008). Serial section scanning electron microscopy of adult brain tissue using focused ion beam milling. J. Neurosci..

[20] Minoda H, Tamai T, Iijima H, Hosokawa F, Kondo Y (2015). Phase-contrast scanning transmission electron microscopy. Microscopy.

[21] Minoda H, Tamai T, Ohmori Y, Iijima H (2017). Contrast enhancement of nanomaterials using phase plate STEM. Ultramicroscopy.

[22] Iijima H (2014). Hybrid fluorescence and electron cryo-microscopy for simultaneous electron and photon imaging. J. Struct. Biol..

[23] Heikal AA, Hess ST, Webb WW (2001). Multiphoton molecular spectroscopy and excited-state dynamics of enhanced green fluorescent protein (EGFP): acid base specificity. Chem. Phys..

